# A Low-Noise, Modular, and Versatile Analog Front-End Intended for Processing *In Vitro* Neuronal Signals Detected by Microelectrode Arrays

**DOI:** 10.1155/2015/172396

**Published:** 2015-04-21

**Authors:** Giulia Regalia, Emilia Biffi, Giancarlo Ferrigno, Alessandra Pedrocchi

**Affiliations:** ^1^Neuroengineering and Medical Robotics Laboratory, Electronics, Information and Bioengineering Department, Politecnico di Milano, 20133 Milan, Italy; ^2^Bioengineering Laboratory, Scientific Institute IRCCS Eugenio Medea, 23842 Bosisio Parini, Italy

## Abstract

The collection of good quality extracellular neuronal spikes from neuronal cultures coupled to Microelectrode Arrays (MEAs) is a binding requirement to gather reliable data. Due to physical constraints, low power requirement, or the need of customizability, commercial recording platforms are not fully adequate for the development of experimental setups integrating MEA technology with other equipment needed to perform experiments under climate controlled conditions, like environmental chambers or cell culture incubators. To address this issue, we developed a custom MEA interfacing system featuring low noise, low power, and the capability to be readily integrated inside an incubator-like environment. Two stages, a preamplifier and a filter amplifier, were designed, implemented on printed circuit boards, and tested. The system is characterized by a low input-referred noise (<1 *μ*V RMS), a high channel separation (>70 dB), and signal-to-noise ratio values of neuronal recordings comparable to those obtained with the benchmark commercial MEA system. In addition, the system was successfully integrated with an environmental MEA chamber, without harming cell cultures during experiments and without being damaged by the high humidity level. The devised system is of practical value in the development of *in vitro* platforms to study temporally extended neuronal network dynamics by means of MEAs.

## 1. Introduction

At the present time, the* in vitro* study of neuronal network electrical activity under physiological or pathological conditions largely relies on Microelectrode Arrays (MEA), which are substrate-integrated extracellular electrode matrices kept permanently in contact with neurons in culture [1–5]. Thanks to the distributed (i.e., ~60–250 electrodes in standard MEAs) and noninvasive character, this well-established technology provides the possibility to perform network-level long-term studies, overcoming conventional* in vitro* electrophysiology techniques (i.e., patch clamp). MEA-based neuronal-electronics interfaces have been shown to facilitate the study of a bulk of neuronal network processes, including network dynamics, network development, learning and memory, short-term and long-term neuronal plasticity, excitotoxicity, effects of pharmacological treatments, and mechanisms underlying pathological conditions [2, 3, 5, 6].

Nowadays, complete systems for the interfacing electronic circuitry (i.e., amplification and filtering) and the acquisition of MEA signals are commercially available to researchers by few principle players on the market (e.g., Multi Channel Systems GmbH, Plexon Inc., Axion Biosystems Ltd., and Alpha MED Scientific Inc.). Nonetheless, commercial solutions do not always meet some demanding needs, such as low power, compactness, compatibility with experimental setup constraints (e.g., size, environmental conditions), flexibility (e.g., easiness to change component values if needed), or cost-effectiveness. For this reason, some researchers have resorted to the utilization of in-house designed MEA interfacing electronics [7–14].

Besides the obvious advantage for battery powered MEA systems [7, 8, 11], a low power MEA interface system is desirable to avoid perturbations to the biological sample caused by the measurement equipment. Indeed, MEA preamplifier stages are usually placed in close proximity to the neuronal cell culture coupled to the MEA substrate to minimize signal attenuation and noise coupling, enhancing the signal-to-noise ratio (SNR) of recordings [5, 15]. This raises the need to limit the amount of produced heat by the circuitry surrounding the array, in order to prevent significant cell culture temperature upward drifts able to perturb neuronal physiology and cell viability (i.e., >38°C) [5, 16]. This issue requires attention mainly in experimental setups integrating climate control capabilities (e.g., portable culturing and recording chamber or cell incubators embedding MEA equipment) to maintain cells viability during prolonged MEA recordings (i.e., >1 hour). Indeed, the encapsulation of the MEA recording equipment in a confined space kept at physiological temperature hinders or slows down thermal dissipation. A common solution to perform climate-controlled recordings is to insert a commercial MEA preamplifier stage (i.e., the MEA1060 device sold by Multi Channel Systems GmbH) inside a cell incubator [17, 18]. However, the power consumption of the MEA1060 (i.e., 2 W) requires the integration of additional devices (e.g., heat sink) in order to neither damage cells due to overheating nor perturb the incubator temperature controller [17, 18], raising possible issues of sterility and encumbrance. Besides the problem of overheating, the performance of MEA equipment integrated in such setups is downgraded by the high level of humidity (i.e., relative humidity > 90%) traditionally used in cell culture environments to maintain osmolarity and thus cell viability. This imposes to lower the humidity to ambient levels (i.e., <60%) in order not to damage MEA interface boards, which however induces a faster osmolarity increase [17–20].

Concerning compactness, the availability of a compact MEA interface hardware eases handiness of the setup, its transfer in the laboratory setting, and the handling of a high number of channels (e.g., when performing parallel recordings from different cultures or from high density arrays). As for what concerns the power consumption, this feature is advantageous in cell incubator MEA setups and even more binding in stand-alone culturing and recording systems [11, 12, 19]. In order to attain the lowest size multichannel interface electronics (i.e., order of mm), an increasing number of laboratories have been resorting to integrated circuits (ICs) [9, 10, 13, 14, 21, 22], together with a few multichannel systems vendors (e.g., Axion Biosystems Ltd., Intan Technologies LLC). However, still the majority of MEA interfacing technologies routinely employed in* in vitro* systems are based on conventional off-the-shelf, discrete components (e.g., Multi Channel Systems GmbH, Alpha MED Scientific Inc., and Plexon Inc.), since the development of ICs requires special in-house facilities and expensive machinery processes and capabilities that overcome those of most researchers [8, 23]. Moreover, the recording noise is often a limiting factor in integrated approaches, since it increases with the decrease of transistor size [3, 5, 14]. Discrete electronic boards facilitate replication by other laboratories, important when developing or progressing prototypal setups [8]. Moreover, by densely arranging component placement and resorting to multilayer printed circuit boards (PCBs), it is possible to achieve overall reasonably limited sizes (i.e., in the order of cm) [7]. Accordingly, custom boards based on discrete components implementing filtering and amplification of MEA signals were described by some groups in the MEA literature [7, 8, 11, 12]. However, these setups lack flexibility, since they embed all the processing stages (i.e., preamplification, filtering, and data conversion) on the same board [7, 12], which does not always permit arranging the electronic parts in a way compatible with the experimental setup constraints [11]. Moreover, existing systems do not present a fully custom analog front-end [8, 11] or they were not designed with attention to customizability (i.e., their employment is confined to the system parts available in the developing laboratory [7, 11, 12]). Furthermore, some of them implement a processing of a limited number of channels per board (e.g., 16 channels per board [7, 8, 18]), which prevents obtaining a good trade-off between compactness and modularity, especially in multi-MEA setups (i.e., standard 64-channel MEAs would require 4 boards each).

The aim of this work is to address the aforementioned need of a modular and versatile MEA interface system and to overcome the issues related to electronics thermal dissipation and humidity damage in MEA setups integrated with cell culturing environments. Accordingly, we introduce a low-noise, modular, and versatile 60-channel MEA front-end including a low power preamplifier. Design requirements and the definition of the overall circuitry, details about the physical implementation of the defined electronics on printed circuit boards, and performance assessment through comparison with benchmark equipment are reported. Moreover, the suitability of the system to be coupled to a stand-alone culturing chamber and to perform stable measurements of neuronal culture activity for long periods is demonstrated.

## 2. Methods

### 2.1. Design Requirements

Features of typical extracellular spikes detected by standard MEAs are (i) amplitude ranging from 30 *μ*V to 1 mV peak-to-peak, (ii) frequency content ranging between a few hundred Hz and a few kHz, (iii) overlapping microelectrode thermal and background biological noise ~20 *μ*V peak-to-peak (~3-4 *μ*V RMS), and (iv) microelectrode offset in the order of few hundred mV [24, 25]. The following requirements have been defined for the processing hardware:

1. bandwidth gain ~1000, to achieve a good signal resolution with standard A/D cards used for MEA signals (input range of few volts and resolution around 14–16 bit),
2. gain as flat as possible over the bandwidth and group delay approximately constant, to minimize waveform distortion,
3. frequency range suitable for extracellular spikes generated by neuronal networks, that is, −3 dB points around 300 Hz and 3 kHz,
4. attenuation of at least 40 dB at the Nyquist frequency compared to the gain in the pass band, to avoid aliasing,
5. tolerance to microelectrode offset, in order not to exceed amplifier maximum input/output ranges,
6. input-referred noise of custom electronics < microelectrode noise, so that the circuit does not degrade input SNR,
7. minimization of the number of components, to reduce board physical occupation,
8. modularity, that is, physical separation between main circuit stages.

### 2.2. Design of Signal Readout Electronics

The signal chain from MEA microelectrode to the data acquisition (A/D) device is composed of a preamplifier stage followed by a filter amplifier stage.

#### 2.2.1. Preamplifier Design

**Figure 1:**
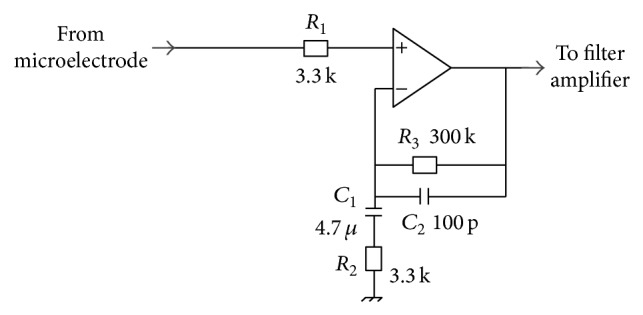
Scheme of preamplifier.

Preamplifier schematic is shown in Figure 1.

*Circuit Topology.* Preamplifier topology exploits a single operational amplifier in a noninverting band-pass filter configuration. This provides a high input impedance, needed to not attenuate signal coming from the high impedance MEA microelectrode (typically, 100 kΩ @ 1 kHz) [21]. Moreover, the circuit exploits only one operational amplifier per channel without inverting signal polarity. Since MEA signals are typically acquired in reference to a bigger electrode immersed in the culture medium and tied to the ground of the system, the preamplifier exploits a single-ended topology. Circuit transfer function, bandwidth gain, and −3 dB lower and upper frequency are provided by (1), (2), (3), and (4), respectively:

(1) $$\begin{array}{c} G_{\text{pre-amp}}\left(s\right)=\frac{1+s\left(C_{1}R_{3}+C_{1}R_{2}+C_{2}R_{3}\right)+s^{2}\left({R_{2}R_{3}C}_{1}C_{2}\right)}{\left(1+sR_{2}C_{1}\right)\left(1+sR_{3}C_{2}\right)}, \end{array}$$

(2) $$\begin{array}{c} K_{\text{pre-amp}}=1+\frac{R_{3}}{R_{2}}, \end{array}$$

(3) $$\begin{array}{c} f_{n\text{-HP}}=\frac{1}{2\pi R_{2}C_{1}}, \end{array}$$

(4) $$\begin{array}{c} f_{n\text{-LP}}=\frac{1}{2\pi R_{3}C_{2}}. \end{array}$$

The preamplifier does not cut the offset (i.e., *G*_pre-amp_  (*s* = *j*2*πf* = 0) = 1), which is cut by the following stage. The gain of the preamplifier should be as high as possible to make the effect of noise introduced by the following stages negligible, thus not affecting SNR [7, 9, 15, 25]. On the other hand, the higher the gain, the higher the noise of the preamplifier, which could exceed the microelectrode noise [26]. A value of *K*_pre-amp_ near 90 has been set as a good trade-off, by means of noise simulations (LT Spice IV, Linear Technology), which is comparable to other proposed preamplifiers [7, 8]. Concerning the bandwidth, *f*_*n*-HP_ = 10 Hz and *f*_*n*-LP_ = 5 kHz have been chosen.

*Components Selection.* The selection of passive component values to achieve the desired gain and bandwidth was performed by manual computations and confirmed by simulations of frequency response gain (Table 1). Through simulations, low-noise operational amplifiers (OPs) (<9 nV/√Hz at 1 kHz) were found to be suitable to fulfill noise constraints. Moreover, low-input offset voltage (<±100 *μ*V) should be preferred to result in a negligible preamplifier output offset contribution compared to electrode offset. Also a small bias current is required not to increase circuit offset, since the dc resistance between the electrode and ground is usually very high (>10^7^ Ω) [27, 28]. Particularly, values in the order of pA should be preferred because they do not cause any electrochemical changes in the electrodes [12]. Finally, a further requirement for preamplifier OP is a low quiescent current (<1 mA) in order to limit power consumption and thus heat production which may harm the cells (as discussed in Introduction). The chosen OP is OPAx376 (Texas Instruments), which is a low noise (7.5 nV/√Hz at 1 kHz), low input offset voltage (5 *μ*V) and input bias current (0.2 pA), low quiescent current (760 *μ*A) OP.

*Power Supply.* Considering a maximum peak-to-peak preamplifier output equal to ~90 mV (1 mV × 90) and a maximum electrode offset equal to ±500 mV [25], the minimum bipolar power supply to avoid saturation of preamplifier outputs should be ±550 mV. However, to minimize the chance of op-amp saturation, a bipolar power supply of ±2.77 V for the preamplifier was chosen, which is reasonably low to reduce noise and power consumption. Positive voltage regulator REG102 and negative voltage regulator TPS72301 (Texas Instruments) were found suitable to provide the required output current to feed the preamplifiers (i.e., 760 *μ*A × 60 channels = ~45 mA).

#### 2.2.2. Filter Amplifier Design

**Figure 2:**
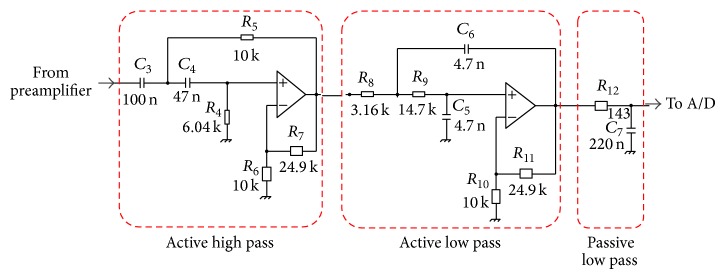
Scheme of the filter amplifier.

Filter amplifier schematic is shown in Figure 2.

*Circuit Topology.* The topology of active filters is the voltage-controlled voltage-source [29], which allows a high input impedance, requires one OP for each pole pair, and does not invert signal polarity. Equations (5) to (7) relate to the 2nd order active high-pass filter transfer function, gain, and natural frequency, depending on component values. Equation (8) underlines the link between the filter damping factor (*ξ*_HP_) and the other filter parameters:

(5) Gactive-HPs=KHPs2·s2+s1R4C4+1R4C3+1−KHPR5C3ppppppppp1R4C4+1R4C3+1−KHPR5C3+1R4R5C3C4−1,

(6) $$\begin{array}{c} K_{\text{HP}}=1+\frac{R_{7}}{R_{6}}, \end{array}$$

(7) $$\begin{array}{c} f_{n\text{-HP}}=\frac{1}{2\pi \sqrt{R_{4}R_{5}C_{3}C_{4}}}, \end{array}$$

(8) $$\begin{array}{c} \frac{1}{R_{4}C_{4}}+\frac{1}{R_{4}C_{3}}+\frac{1-K_{K_{\text{HP}}}}{R_{5}C_{3}}=2\xi_{\text{HP}}2\pi f_{n\text{-HP}}. \end{array}$$

Equations (9) to (11) relate to the 2nd order active low-pass filter transfer function, gain, and natural frequency, depending on component values. Equation (12) underlines the link between the filter damping factor (*ξ*_LP_) and the other filter parameters:

(9) Gactive-LPs=KLP1R8R9C5C6 ·s2+s1R8C6+1R9C6+1−KLPR9C5pppp1R8C6+1R9C6+1−KLPR9C5+1R8R9C5C6−1,

(10) $$\begin{array}{c} K_{\text{LP}}=1+\frac{R_{7}}{R_{6}}, \end{array}$$

(11) $$\begin{array}{c} f_{n\text{-LP}}=\frac{1}{2\pi \sqrt{R_{8}R_{9}C_{5}C_{6}}}, \end{array}$$

(12) $$\begin{array}{c} \frac{1}{R_{8}C_{6}}+\frac{1}{R_{9}C_{6}}+\frac{1-K_{\text{LP}}}{R_{9}C_{5}}=2\xi_{\text{LP}}2\pi f_{n\text{-LP}}. \end{array}$$

Finally, (13) relate to the passive low-pass filter transfer function and natural frequency, depending on component values:

(13) $$\begin{array}{c} G_{\text{RC}\_\text{LP}}\left(s\right)=\frac{1}{sR_{12}C_{7}+1},\\ f_{n\text{-RC}\_\text{LP}}=\frac{1}{2\pi R_{12}C_{7}}. \end{array}$$

The −3 dB points of active filters are determined by (7) and (11), provided the active filters are Butterworth filters (i.e., *ξ*_HP_ and *ξ*_LP_ equal 0.7071), which maximizes gain flatness [29]. 

*Component Selection.* Setting *K*_HP_ = *K*_LP_ = 3.5, *f*_*n*-HP_ = 300 Hz, and *f*_*n*-LP_ = *f*_*n*-RC_LP_ = 5000 Hz resulted in the component values reported in Table 2. Given the cascade between the active low pass and the RC (*ξ* = 1), the resulting attenuation at 5000 Hz is 6 dB.

In order to avoid loading the filter, the circuitry must guarantee that the impedance of sources to the filter is low and the impedances of sinks are high. The output impedance of the preamplifier stage is <10 Ω (manufacturer's specifications), which is much lower than the input impedance of the high-pass filter. Similarly, the input impedance to the A/D card is usually bigger than 1 kΩ, which is much higher than passive low-pass filter's *R*_12_. The utilization of an additional OP to act as a buffer before the A/D device was avoided, as done in [8], thus limiting the PCB size.

Though the higher contribution to noise comes from the preamplification stage, the selection of OPs for the filter amplifier stage was also led by the constraint of low noise. Moreover, since at the chain output the dc component of the final low-pass stage is present, the OP should also introduce a low offset. The defined operational amplifier for both the active and low-pass filter is OPx177 (Texas Instruments), which is a low-noise (8 nV/√Hz at 1 kHz), low-offset (<60 *μ*V), and low-input bias current (2 nA) operational amplifier. 

*Power Supply.* Given a maximum filter amplifier peak-to-peak output around 1.1 V (1 mV × 1100) and a negligible dc output (i.e., offsets of the active low-pass filter), the minimum power supply for the filter amplifier should be around ±550 mV. However, to exploit an available power supply, a bipolar supply voltage of ±7 V for the filter amplifier was chosen. Even though this results in an increase of the noise level of filter amplifiers, input-referred noise of filter amplifier is negligible compared to preamplifier output noise level, as confirmed by simulations (i.e., ~1.5 *μ*V RMS versus ~70 *μ*V RMS).

#### 2.2.3. Overall Response

The overall response provides a gain of ~1100, similar to other circuits in the MEA literature [9, 12, 14, 21, 28] and in commercial devices (Multi Channel Systems GmbH, Axion Biosystems Ltd.). The whole frequency band is delimited by 2 high-pass poles (i.e., slope equal to +40 dB/decade) and 4 low-pass poles (i.e., slope equal to −80 dB/decade). The −3 dB point of the entire system can be calculated from the product of each stage's transfer function. This yields systemwide −3 dB points at 300 Hz and 3.03 kHz, which respects the bandwidth requirements. The frequency of 12 kHz is attenuated by 40 dB compared to the frequencies in the pass band, which suggests a minimum sampling frequency of 25 kHz, as commonly done with MEA recordings [25]. A 50 Hz signal at the preamplifier input is amplified by a factor of 10, which is 40 dB less (i.e., 100 times less) than the gain in the pass band. Further attenuation of the 50 Hz interference is typically left to digital filters. These conclusions were verified in a LT Spice simulation which included input impedance of A/D device and impedance of the cables connecting the preamplifier with the filter amplifier and the filter amplifier with the A/D device. Cables were modeled as low-pass filter with *R* = 5 Ω and *C* = 128 pF. The simulated overall RMS input noise, obtained by dividing the output noise by the nominal gain [30], is around 0.65 *μ*V RMS, corresponding to ~4 *μ*Vpp [24], fulfilling noise constraints.

### 2.3. Realization of Printed Circuit Boards and Assembly

#### 2.3.1. Board Layout

**Figure 3:**
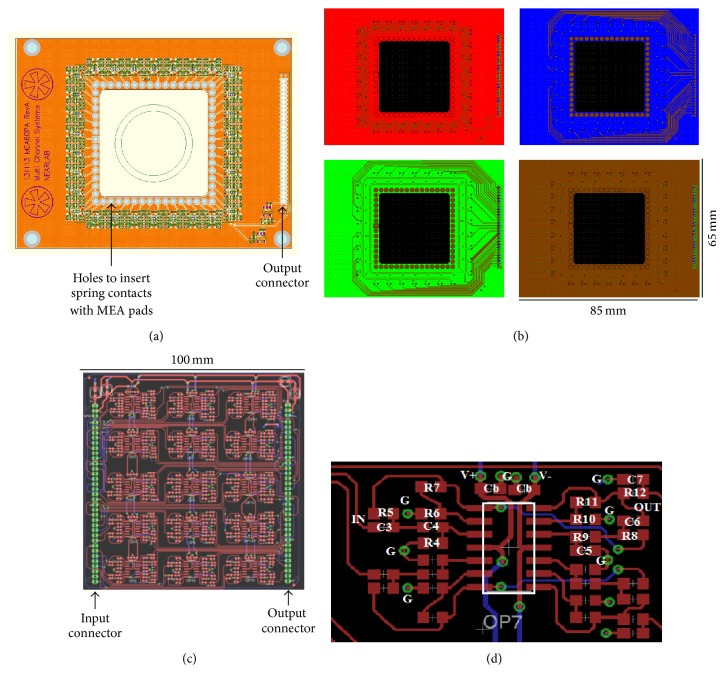
(a) Top view of the 60-channel preamplifier board rendering, highlighting the position of the holes for the insertion of miniaturized spring contacts connecting MEA pads and preamplifier inputs and the location of the output connector. (b) Top view of the four layers composing the preamplifier PCB. Red = top layer, with components and ground plane; blue = mid layer 1, with traces for output signals; green = mid layer 2, with traces for output signals and power supply traces; brown = bottom layer, with ground plane. On the right of each layer there are double row connectors for output signals. (c) Top layer of the dual layers filter amplifier PCB, highlighting signal and power supply traces and components (in red) and signal input/output connectors (in green). The bottom layer (not shown) is dedicated to the ground plane. (d) Detail of the disposition of passive components around each quad-OP of the filter amplifier boards. Component names refer to those in Figure 2. G = ground and Cb = bypass capacitors on power supply lines (V+ and V−).

**Figure 4:**
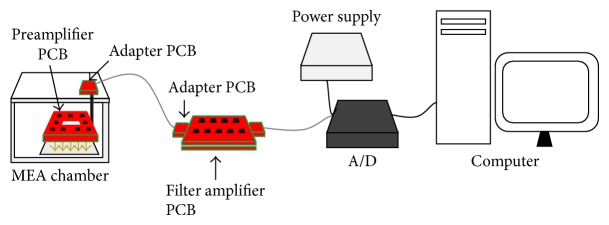
Scheme of the assembled setup showing the disposition of the custom preamplifier board (inside a PMMA-made MEA chamber), the custom filter amplifier board, the A/D device, and the power supply (Multi Channel Systems GmbH). The preamplifier PCB contacts the MEA pads by means of gold spring contacts (schematized by the gold arrows).

The boards were manufactured using standard PCB technology. The preamplifier board has been designed with Altium Designer (Altium Ltd.), obtaining a four-layer layout (Figures 3(a) and 3(b)). The size of the board has been set to 65 × 85 mm. A squared 33 × 33 mm hole has been located in the center of the board, matching the size of standard glass or plastic culture containers coupled to MEA substrates (Multi Channel Systems GmbH). Along the edge of the hole, 60 miniaturized gold spring contacts (RS Components Ltd.) have been inserted through appropriate holes to vertically contact the MEA pads by pressure (Figures 3(a) and 4). The presence of the hole ensures the maximum physical proximity between pads and electronics. Operational amplifiers were chosen with a miniaturized, dual channel surface mounted package (OPA2376, M-SOP8 package), while passive components package was set to 0201, to reduce the physical occupation of the parts. On the top layer (red in Figure 3(b)), components (preamplifiers and voltage regulators) and ground plane were located; on the layer beneath (blue) traces for half the output signals were drawn, while the remaining ones were put on the other middle layer (green), together with the power supply traces; the bottom layer (brown) was filled with a ground plane. Output signals and power supply traces are organized in a 64-pin, 1.27-pitch, double row output connector (Figure 3(a)).

The filter amplifier board layout was designed with software EAGLE (CadSoft). Two identical 100 × 100 mm dual layer 30-channel boards were designed (Figure 3(c)) in order to be stacked one above the other. Components, signal traces, and power supply traces were placed on the top layer, while the ground plane is on the bottom layer. To reduce physical occupation, quad-OPs (OPA4177, SO-14 package, Texas Instruments) and 0603 passive components have been chosen. Each quad-OP has been dedicated to implement the band-pass filters for two MEA channels. The layout was designed in order to assure an identical disposition of passive components around each quad-OP, as shown in the zoom of Figure 3(d).

#### 2.3.2. Assembly of the Setup

The experimental setup exploited to test the performances of the system is depicted in Figure 4. The preamplifier board was inserted inside a polymethyl methacrylate (PMMA) environmental chamber (similar to the one described in [19]), able to maintain incubator-like conditions (i.e., temperature at 37°C, relative humidity >90%, and 5% CO_2_). To avoid damage due to the high humidity level, the board was sheltered with an ad hoc polylactic acid (PLA) cover and sealed by biocompatible silicon. Then, it was fixed to the top plate by means of grounded screws on each corner (Figure 3(a)), in order to assure a vertical alignment between the contact pins and MEA pads. The filter board was placed on the outside.

To connect preamplifier and filter amplifier board, we exploited an available 64-pin shielded cable (MCS68 cable, Multi Channel Systems GmbH) and connected both ends to custom PCB adapters compatible with preamplifier output connector and filter amplifier input connector. The power supply for both boards is delivered by a commercial device (PS40W, ±7 V, 40 W, Multi Channel Systems GmbH). The device has been directly connected to the filter board power supply connectors. To deliver the power supply to preamplifiers, the power lines pass through the 64-pin cable linking the two boards and are lowered to ±2.77 V by the on-board voltage regulators. Filter outputs have been connected to an available A/D card (16 bit, ±4 V, up to 50 kHz/channel, USB-ME64, Multi Channel Systems GmbH), by means of a second cable. Digitized signals can be visualized and stored with software McRack (Multi Channel Systems GmbH). The ground of the MEA recording system was connected to that of the acquisition computer and the chamber environment control system, in order to prevent ground loops.

### 2.4. Tests to Evaluate Board Performances

Measurements of circuit noise were performed connecting all inputs to the ground of the system and measuring circuit outputs. To obtain input-referred values, voltage output was divided by the overall bandwidth gain of the circuit. Then, signal power spectral density (Welch periodogram) was integrated over the range of neuronal spikes (300 Hz and 3 kHz) and the squared root was extracted to obtain root-mean-squared noise levels (RMS) [9, 19]. To characterize the setup noise magnitude when the circuit is connected to MEA electrodes, inputs were connected to a MEA (200/30iR, Multi Channel Systems GmbH) filled with phosphate buffered saline [31]. The same chips were put in a commercial recording device (USB-ME64 system, composed of the MEA1060 preamplifier and the FA64 filter amplifier, overall gain, 1100, bandwidth, 10 Hz–3 kHz, Multi Channel Systems GmbH) to compare noise levels. A *t*-test for paired samples (*p* < 0.05) was performed for each MEA recorded in the two systems (*n* = 3).

Cross talk was measured by sending a 1 mV 1 kHz voltage-controlled sine wave to one channel (driving channel) and recording from adjacent or nonadjacent channels, whose inputs were tied to ground, as done in [31]. Cross talk gain was defined by the ratio of the grounded channel output to the grounded channel input. Channel separation was computed as the ratio between the driving channel output and the grounded channel output.

In order to evaluate the frequency-dependent behavior of the devised front-end, sinusoidal waveforms (peak-to-peak amplitude, 100 *μ*V, frequency, 1 Hz–20 kHz) were generated with a wave generator (TG315, Thurlby Thandar Instruments) and provided as inputs to each of the preamplifiers. For every frequency, the Fast Fourier Transform (FFT) of input and output was then computed. Output FFT was divided by input FFT to obtain the gain and the phase shift of the circuit.

The power consumption of each board was measured connecting them to a bench-top power supplier measuring supply current (GPS-4303, Linear Technology).

Finally, the custom boards were connected to MEA chips (*n* = 4) coupled to hippocampal neuronal cultures (obtained and fed as described in [32]). After a waiting time (5 min) to let the cultures accommodate once they were positioned in the setup [33], each MEA was recorded for a few minutes. After digital signal filtering (300 Hz–3 kHz, Butterworth 2nd order), spikes were detected comparing voltage values with a threshold appointed to −5 times the standard deviation of signal computed in the first 500 ms of recording [34]. Then, the SNR of firing electrodes was computed as the ratio of the peak-to-peak amplitudes of spikes by the standard deviation of signal computed over the first 500 ms. The same cultures were recorded in the benchmark setup to compare SNR values. Measurements of the same MEAs in the two setups were performed right sequentially, in order to avoid any change in SNR due to network activity change along with maturation or alteration of cell-electrode coupling or biological background noise magnitude. After assessing nonnormality of the data (Shapiro Wilk test), Wilcoxon-matched paired test (*p* < 0.05) was applied to compare SNR values referred to the same channels of a MEA in the two setups. In all the tests, output signals were acquired with McRack software and processed with custom scripts in Matlab (The Mathworks).

## 3. Results

### 3.1. Realized Boards

**Figure 5:**
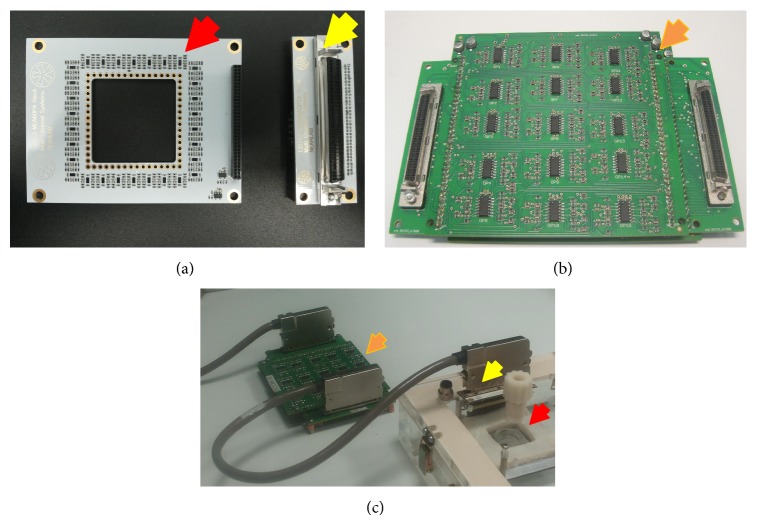
(a) Picture of the realized preamplifier board (red arrow) and adapter board (yellow arrow) which organizes output signal in a 64-pin socket. (b) Picture of the realized filter amplifier, constituted of two identical stacked 30-channel PCBs, with adapters boards for cables plug-in on both sides. (c) Assembly of the board in the experimental setup. The preamplifier (red arrow) contacts MEA electrodes inside a custom-built chamber and is protected against high humidity level through a PLA cover. Signals are sent to the adapter on the top plate (yellow arrow) and then to the filter amplifier (orange arrow) through the shielded cable.

The realized and assembled boards are depicted in Figure 5(a) (preamplifier on the left and PCB adapter for the cable plug-in on the right) and Figure 5(b) (two 30-channel filter amplifier stacked PCBs).  Figure 5(c) represents the assembly of the boards with the MEA chamber (described in Section 2.3.2), showing the internal board protected against high humidity level through the PLA cover.

### 3.2. Performance of the Boards

The boards performance is summarized in Table 3.

#### 3.2.1. Noise and Cross Talk

The noise of the circuit was equal to 0.95 ± 0.05 *μ*V (mean ± standard deviation across channels) in the whole hardware bandwidth (i.e., up to half the sampling frequency) and to 0.8 ± 0.04 *μ*V RMS in the spike bandwidth (i.e., 300 Hz–3 kHz), in good agreement with simulations (i.e., 0.65 *μ*V RMS). The broad-band noise level is comparable to those reported previously for other custom amplifiers for extracellular recording over a similar bandwidth [7] or even lower [11, 21, 31]. Moreover, the noise level over the spike bandwidth compares well with the level of the benchmark equipment we used, that is, 0.8 *μ*V RMS (manufacturer's specifications), and is lower than other commercial devices (e.g., The Muse Amplifier sold by Axon Biosystems and the RHD2000 amplifier sold by Intan Technologies LLC) and custom circuits [13]. Noise values of electrodes and electronics (Table 4) were higher than circuit noise, indicating that the circuit adds a negligible noise contribution to the input thermal electrode noise. These values were comparable between the custom board and the commercial system (*t*-test, *p* > 0.05 for all the comparisons).

Several channels were surveyed for cross talk measurements. For directly adjacent channels (i.e., adjacent input pins and same dual-OP in preamplifier boards), the observed cross talk gain was −17 dB at 1 kHz. Considering an extracellular potential of an extremely large magnitude (i.e., 1 mV) at the input of a channel, the system cross talk would result in an output of the coupled channel equal to 0.15 mV, corresponding to an apparent potential of only 0.14 *μ*V at the input of the coupled channel. This value is far below the input-referred noise of the circuit. Relative to the driven channel, channel separation was 77 dB, which is higher than the one measured from other circuits proposed for multichannel recordings with MEAs [11, 12, 31]. Cross talk gain was slightly lower (i.e., higher channel separation) for channels with adjacent input pins but not connected to the same OP, that is, −18 dB (channel separation, 78 dB). Finally, for nonadjacent channels, the cross talk gain was −27 dB and the channel separation 87 dB.

#### 3.2.2. Frequency Behavior

**Figure 6:**
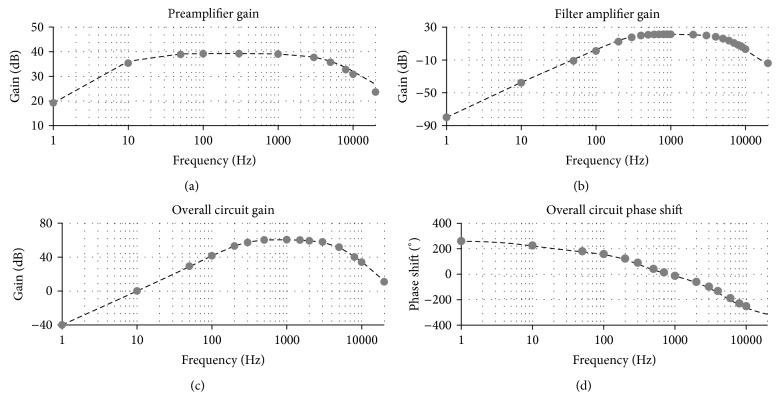
Frequency dependence of the simulated (dashed line) and the measured (gray dots) circuit behavior. (a) Preamplifier gain. (b) Filter amplifier gain. (c) Gain of the overall signal chain. (d) Phase shift of the overall signal chain. Measured data are reported as mean values across 60 channels.

Figure 6 shows the measured preamplifier, filter amplifier, and overall chain gains (Bode diagram), averaged over 60 channels and superimposed to the simulated response. In the spike bandwidth (300 Hz–3 kHz), the mean error is equal to 2.11%, 4.21%, and 2.4%, respectively. The −3 dB corner frequencies of all circuit are at 315 Hz and 3110 Hz, which matches with system design calculations and simulations. Differences between the measured and predicted values of the gains and pole locations can be attributed to tolerances of capacitors and resistors and did not prevent the fulfilment of design requirements.

**Figure 7:**
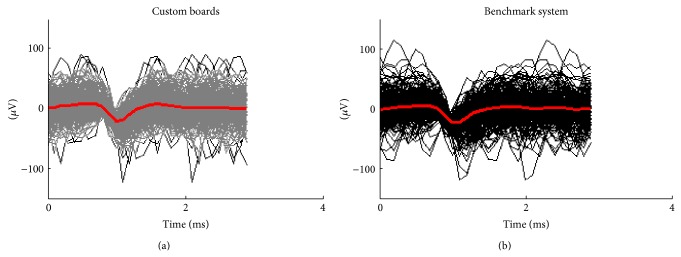
Example of superimposed spike waveforms extracted by the same microelectrode coupled to a culture recorded with the custom board (gray) and with the commercial system (black). Temporal average of waveforms is in red.

Concerning the phase shift, it was equal to −8.4° at 1 kHz, comparing well with the predicted value (i.e., −11°). The phase shift profile was rather linear in the bandwidth (*r* ^2^ = 0.9, Figure 6(d)), making the group delay (i.e., derivative of the phase with respect to the frequency) nearly constant, compared to other circuits for extracellular neuronal potentials [7]. This ensures that the fidelity of the signal is acceptably preserved [7] and allowed for observation of waveforms comparable to the benchmark system (Figure 7). The maximum group delay at any frequency is 700 *μ*s (300 Hz), matching the simulated response.

#### 3.2.3. Signal Quality

**Figure 8:**
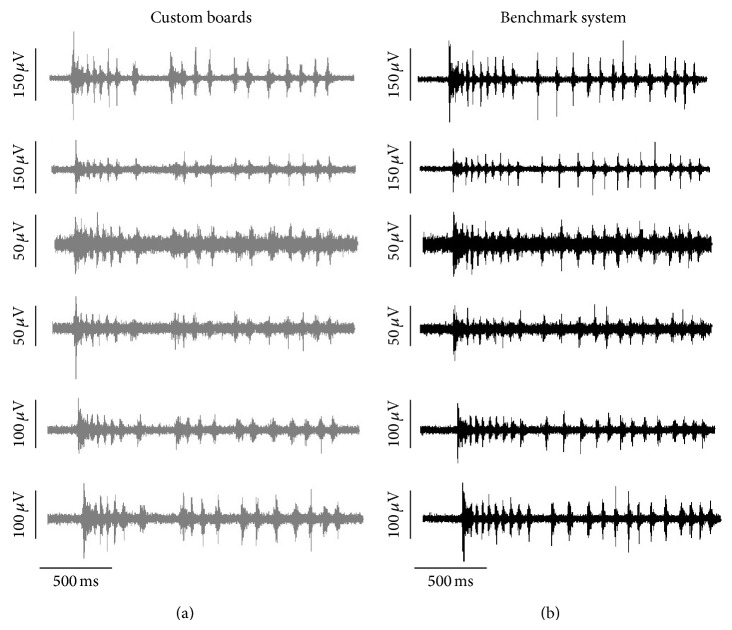
Example of signals detected by the same microelectrodes of a MEA recorded with the custom boards (gray (a)) and with the commercial benchmark system (black (b)).

With the realized boards, it was possible to record extracellular signals from hippocampal cultures satisfactorily comparable with those recorded by the benchmark MEA system. An example of this is provided in Figure 8, where the spiking signals sensed by the same microelectrodes in the custom setup (gray) or in the commercial one (black) are reported. Differences in background noise levels and spike amplitudes at different sites are matched in the two setups.

**Figure 9:**
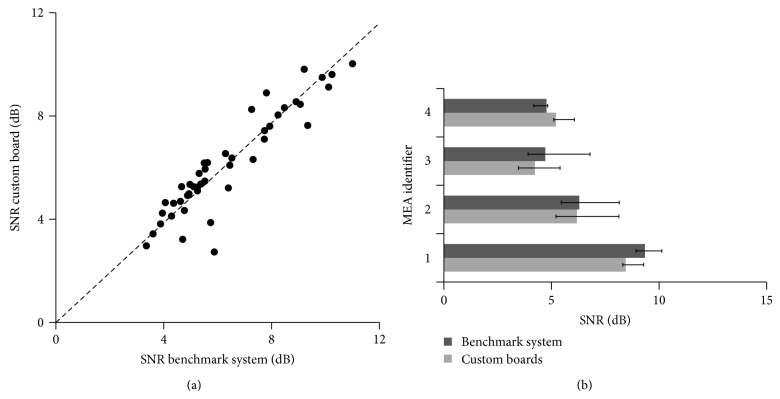
(a) Scatter plot representing SNR values (dB) of electrodes recorded with both the benchmark system (*x*-axis) and the custom boards (*y*-axis). *N* = 46 electrodes extracted by 4 MEA chips. SNR are median values over the spikes detected at each electrode. Dashed line, bisector of the graph, indicating the localization of points in the ideal case of identical SNR values. (b) Histograms of SNR values of MEA signals collected from hippocampal cultures on MEAs (*n* = 4) recorded with the custom board (light gray) and with the commercial system (dark gray). Histograms are reported as median SNR over the spiking channels, with bars spanning from the 25th to the 75th percentile. For all the pairs, SNR values were not statistically different (*p* > 0.05, Wilcoxon matched pair test).

The comparison of signal SNR values related to the two setups revealed similar values, as shown in the scatter plot of Figure 9(a), which links the SNR values measured in the two setups for each of the firing electrodes found present in 4 cultures coupled to MEAs cultures. The projection of most electrodes falls near the bisector line, indicating similar values. For each culture, the statistical analysis did not provide significant differences between SNR values in the custom and the commercial setup (Wilcoxon-matched paired test, *p* > 0.05), as reported in Figure 9(b).

#### 3.2.4. Feasibility of Prolonged Recordings in an Incubator-Like Environment

In addition to ensuring good practice specifications in terms of gain, bandwidth, and noise, the realized MEA interface system has been proven to be compatible with a fully incubator-like environment in terms of both temperature and relative humidity, differently from available commercial solutions previously used to perform MEA recordings inside cell incubators (i.e., MEA1060 system [17, 18] and Muse System [20]), as detailed in Table 5.

Measurements of the power consumption reported a total value (preamplifier and filter amplifier boards) of ~1.4 W. Particularly, the power consumption of the preamplifiers surrounding the MEA (i.e., ~550 mW) was much lower than the one of the MEA1060 preamplifier (i.e., ~2 W, by manufacturer's specification), thanks to the lower voltage supply (i.e., ±2.7 versus ±6–9 V) and to the utilization of OPs with a lower quiescent current (i.e., for 60 channels, ±45 mA versus ±150 mA). This allowed for obtaining a negligible temperature increase once the preamplifiers were enclosed in the environmental chamber (*T* = 37°C). Indeed, temperature increases by less than 1°C (i.e., 0.7°C) after preamplifier switch-on, reaching a stable profile in around 20 minutes.

**Figure 10:**
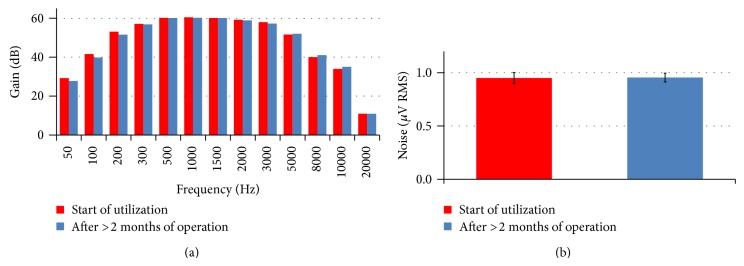
(a) Comparison of the gain of the circuit measured at the beginning of the utilization of the MEA interfacing boards (red) and after more than 2 months of operation in the environmental chamber (blue), at high relative humidity level. Values are averaged across 60 channels. (b) Comparison of input-referred noise of the circuit measured at the beginning of the utilization of the interface boards (red) and after more than 2 months of operation in the environmental chamber (blue). Data are shown as mean and standard deviations across 60 channels (*p* > 0.05, Wilcoxon-matched paired test).

The device was demonstrated to be resistant to the high humidity level required to maintain culture medium osmolarity, differently from incubator setups employing commercial preamplifiers (as mentioned in Introduction and shown in Table 5). Indeed, even after several weeks of continuous operation (i.e., boards left switched on inside the incubator-like environment), we did not observe the uprising of short-circuits or contacts/tracks oxidation, thanks to the effective board sealing. Accordingly, the performances of the system did not undergo modifications over time, neither in terms of frequency-dependent behavior nor in terms of circuit noise, as shown in Figures 10(a) and 10(b), respectively.

**Figure 11:**
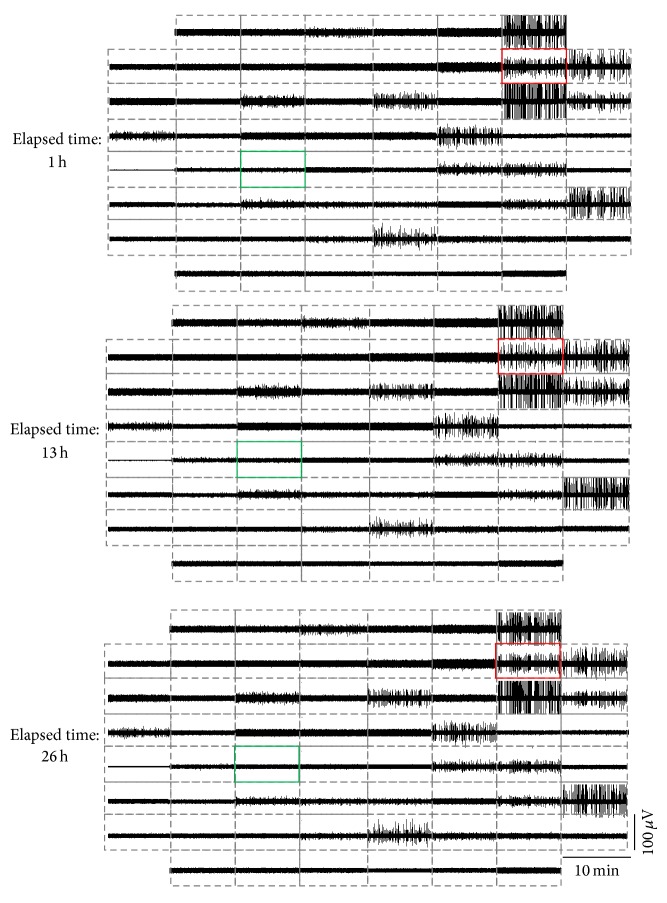
Example of a continuous measurement for several hours performed with the devised MEA interface system coupled to an environmental chamber to preserve cell viability. The three snapshots represent the 60-channel raw voltage activity over 10-minute segments relative to three different moments during the recording, that is, after 1, 13, and 26 hours (as indicated on the left). Green frame, silent channel (only background noise). Red frame, spiking channel.

**Figure 12:**
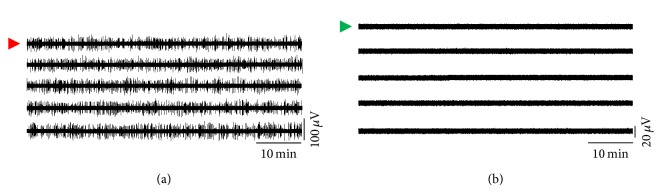
Continuous 5-hour signals collected from two different channels of the MEA recording reported in Figure 11. (a) Raw voltage signal collected by a spiking channel (i.e., extracellular spikes superimposed to biological, electrode, and electronics noise). (b) Raw voltage signal collected by a silent channel (i.e., biological, electrode, and electronics noise).

Further, recordings of neuronal activity performed for several hours did prove the mechanical stability of the preamplifier interface and the absence of baseline drifts and of increasing noise levels. As an example of this, Figure 11 reports three 10-minute snapshots of the 60-channel raw voltage traces detected by a neuronal network monitored for more than 24 hours with the system, showing the multichannel data stability at different time points during the experiment. Voltage measurements at a spiking site (i.e., red in Figure 11) and at a silent site (i.e., green in Figure 11) are depicted in Figure 12.

### 3.3. Versatility of the System

**Figure 13:**
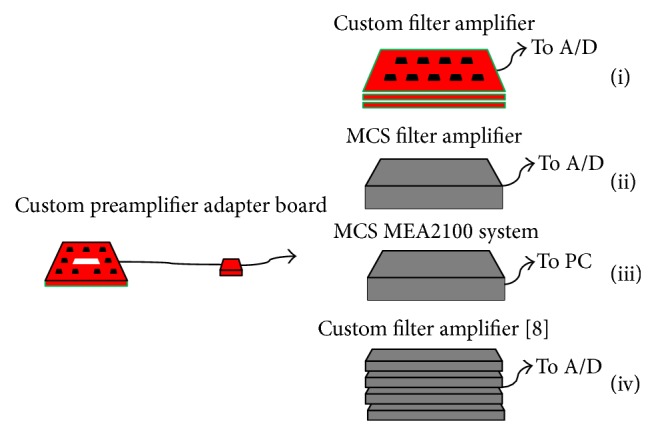
Example of possible arrangements employing components of the here described custom MEA front-end and commercial (MCS GmbH) or custom pieces of equipment. (i) Custom preamplifier and custom filter amplifier developed in our laboratory; (ii) custom preamplifier and MCS filter amplifier; (iii) custom preamplifier and MCS MEA2100 device; (iv) custom preamplifier and custom filters previously proposed [8].

The proposed analog front-end is constructed in two independent, physically separated stages: preamplifier and filter amplifier boards. Thanks to this modularity, portions of our setup can be combined with that of other systems, with the possibility to exchange component values according to the application and to exploit pieces of equipment already available to laboratories, as shown in Figure 10. As an example, we have interfaced our custom preamplifier with Multi Channel Systems hardware for* in vitro* recordings. Specifically, we connected the preamplifier board to the FA64 device (configuration (ii) in Figure 10) and to the MEA2100 system (filter amplifier and A/D, configuration (iii) in Figure 10). Both devices present a broader bandwidth, which would allow for processing MEA signal components different from spikes (i.e., local field potentials). This is possible thanks to the broader bandwidth of our designed custom pre-amp compared to the overall custom chain bandwidth. Measurements show that the cascade of preamplifier boards to FA64 is characterized by an input noise level of 3.6 *μ*V peak-to-peak, comparable to the fully custom front-end (since the main noise source is the pre-amp stage). Concerning configuration (iii), the connection to the MEA2100 system is possible if the pre-amp board gain is lowered (i.e., to 5), due to the restricted input range of the device, which was performed by simply changing values to two components (i.e., *R*_3_ and *C*_2_ in Figure 1). The resulting measured input-referred noise is 7 *μ*V peak-to-peak, still below the electrode noise. Even though we tested the connections to commercial devices from Multi Channel Systems, the connection of preamplifiers to other systems components would be rather simple to implement by means of appropriate adapter boards. Indeed, the preamplifier boards were designed with standard output ranges, so as to exploit possible equipment already available in laboratories using MEAs. For instance, the pre-amp could be easily connected to other open-source design filter amplifiers, like the one designed by Rolston and colleagues [8], as shown in configuration (iv) of Figure 13.

## 4. Discussion

In this work, we thoroughly detailed the design and tested the functioning of a fully custom and modular front-end to collect extracellular signals with MEA intended to be used in experiments in the field of* in vitro* neuroscience. Compared to commercial devices well-established in MEA-based research, main strengths of the system are modularity, compactness, low power consumption, and resistance to high humidity levels, which are advantageous in the development of prototypal setups, such as stand-alone MEA chambers [19], or in the customization of existing equipment, like the case of setups integrated inside cell incubators [17, 18, 20].

The defined circuitry was specifically designed to process and enhance spike waveforms, which represent inputs for MEA data analysis in the vast majority of studies involving MEAs [35]. Accordingly, the hardware bandwidth was fitted to the typical spike waveforms frequency content, discarding out-band noisy components. Compared to broader band acquisition systems (e.g., [8, 11]), this configuration reduces overall noise and does not require to utilize successive digital filters to remove lower frequency components before spike detection.

Minimization of the number of components, without affecting circuit performance in terms of selectivity to spike waveforms, was considered a chief design requirement. The achieved configuration makes use of three amplifiers for each channel, with the gain response acceptably sharp at the bandwidth boundaries (+40 dB/decade and −80 dB/decade). The number of amplifiers per channel is lower than most circuits proposed in the literature [7, 11].

The designed preamplifier stage is not AC coupled to MEA electrodes; thus, the electrode offset is eliminated by the following stages. An alternative topology used in MEA circuits connects the high impedance microelectrode to the noninverting OP input by means of a passive RC high pass, to cut the offset before the preamplification [7, 27]. However, this would require a high *R* value (*R* ≫ 100 kΩ) in order to allow the maximum voltage transfer from the microelectrode to the preamplifier [31], resulting in a higher noise level. Buffers interposed between the microelectrode and the RC and between the RC and the preamplifier to solve this problem would increase the number of components and the noise level; thus, this alternative was avoided.

The utilization of a cascade of Butterworth filters allowed for obtaining the maximum flat possible gain response, which is important to preserve waveforms fidelity. In contrast, other MEA signal processing circuits exploit Bessel filter in order not to alter the shape of neuronal spikes (e.g., [7, 11]). Bessel filters minimize phase distortion at the expense of sharp filter roll-offs, so higher order filters must be used [7]. Since an important constraint in our project was the minimization of the number of circuit components per channel, in order to reduce sizes and costs, we resorted to the Butterworth configuration. Nonetheless, the circuit allowed for extraction of spike waveforms comparable to the commercial benchmark system from Multi Channel Systems GmbH (MEA1060).

The defined topology was demonstrated to stick to all the design requirements. Thanks to the selection of low-noise amplifiers, noise levels were comparable to the benchmark system. Moreover, we achieved an electronics noise level comparable to or better than other proposed custom front-ends for extracellular signals [7, 11, 13, 21, 31]. Furthermore, recordings of neuronal extracellular activity with a SNR comparable to the benchmark device were obtained. The comparability of SNR values ensures comparable performances of a spike detector exploiting a noise level-based threshold, as commonly done to detect spikes from MEA signals [32, 34].

The defined board layout was designed having in mind channel symmetry and compactness as chief features. The achieved physical occupation of the boards was satisfactory. Indeed, preamplifier boards could be easily fitted to a bench-top MEA chamber (as shown in Section 3.1). Moreover, filter amplifiers occupy an area similar to commercial filter amplifier devices processing the same number of channels (e.g., FA64 MCS GmbH). The number of channels processed by each module (i.e., 60) is equal to standard MEA chips electrode number, which is advantageous especially for multi-MEA setups because it reduces the number of identical boards to pile up (differently, e.g., from the filter amplifiers proposed by [8, 11]). Both the preamplifier and the filter amplifier layout include components on one side of the boards, in order to reduce the overall cost and take advantage of large copper filled planes. Indeed, ground planes reduce inductive and capacitive coupling between closed signal traces and help guarding against external electromagnetic interferences, so that special shield is not mandatory in our setup, differently from other systems [7, 8]. Nevertheless, the integration of an* ad hoc* shielding system in order to guard against interferences arising in different laboratory settings is being considered as a future task.

The realized modular boards were successfully integrated in a bench-top chamber similar to the one previously devised by our group [19]. Previous attempts to process MEA signals with a first amplification stage outside the chamber had shown a significantly lower signal quality, due to the absence of impedance decoupling and signal amplification in close proximity to the electrodes [19]. This issue has been solved in this work and the chamber integrated to the devised front-end is currently being exploited to perform MEA recordings. Thanks to the limited power consumption of the preamplifiers compared to the benchmark commercial system, heat dissipation does not add a significant contribution to the temperature in the chamber and does not compromise temperature stability. This allows for avoiding an extra heat-exchange device to remove excess heat produced by preamplifiers, differently from other setups enclosing commercial preamplifiers inside cell incubators [18]. A further reduction of preamplifier board consumption would be easily achieved by decreasing the voltage input to the preamplifier voltage regulator. Besides showing that the presence of the boards does not alter the environmental conditions, we proved that the incubator-like environmental conditions did not undermine board performances. Indeed, as shown in Section 3.2.4, the system was proven not to be damaged over time in spite of the presence of an incubator-like humidity level, which allows for preservation of standard conditions of culture practice when performing recordings, differently from incubator setups [17, 18, 20].

## 5. Conclusions

The presented custom MEA interface system was demonstrated to properly process extracellular neuronal spikes, with functional performances comparable to the benchmark* in vitro* MEA equipment. The design here proposed and thoroughly detailed may be a useful reference in the research field centered on the development of novel MEA-based systems. Indeed, its modularity makes it possible to easily insert any component of this system in a MEA experimental setup, with advantages in applications requiring customizability. For instance, its features would allow for the use of the system for applications exploiting cell-based biosensors, portable neuronal chambers, or setups inside cell incubators. The practical advantage of the front-end was demonstrated by successfully coupling it to a portable neural signal recording station integrated with environmental control systems. Thanks to the compatibility with the incubator environment, this system lends itself to be employed for the monitoring of temporally extended (>hours)* in vitro* neuronal network dynamics. Future work will be focused on the integration of an electrical stimulation system and on the replication of the devised system to perform parallel recordings from different cultures coupled to MEAs.

## Figures and Tables

**Table 1:** Values of preamplifier circuit components. Resistor values (*R*) are in [Ω] and capacitor values (*C*) are in [F]. Components' names refer to Figure 1.

*R*1	*R*2	*R*3	*C*1	*C*2

3.3 k	3.3 k	300 k	4.7 *μ*	100 p

**(a):** 

Active high pass filter					
*R*4	*R*5	*R*6	*R*7	*C*3	*C*4
6.04 k	10 k	10 k	24.9 k	100 n	47 n

**(b):** 

Low pass filter (active + RC)							
*R*8	*R*9	*R*10	*R*11	*R*12	*C*5	*C*6	*C*7
3.16 k	14.7 k	10 k	24.9 k	143	4.7 n	4.7 n	220 n

**Table 3:** Summary of board performances versus predicted values.

Parameter	Measured value	Predicted value
Gain at 1 kHz	1052	1053
*f* −3 dB (low)	315 Hz	300 Hz
*f* −3 dB (high)	3110 Hz	3030 Hz
Group delay (maximum)	700 *μ*s	700 *μ*s
Noise (input referred, spike bandwidth)	800 nV RMS	650 nV RMS
Power consumption (pre-amp)	552 mW	600 mW
Power consumption (filter amp)	826 mW	756 mW

**Table 4:** Comparison of noise levels between custom and commercial benchmark equipment.

[*μ*V RMS]	Custom board	Benchmark system
MEA 1	2.08 ± 1.01	1.97 ± 0.71
MEA 2	1.70 ± 0.24	1.88 ± 0.15
MEA 3	2.01 ± 0.34	1.98 ± 0.27

Input-referred RMS noise levels (mean ± std, *n* = 60 channels) measured connecting 3 MEA chips filled with phosphate buffered saline to the custom board and to the commercial benchmark recording equipment. Noise levels refer to 300 Hz–3 kHz frequency range and were comparable between the two setups.

**Table 5:** Comparison of main specifications of the proposed system and two commercial products.

Parameter	This work	MEA1060	The Muse System
		(Multi Channel Systems GmbH)	(Axion Biosystems Ltd.)
Gain	1052	1100^**^	1200
*f* −3 dB (low)	315 Hz	300 Hz^*^	200^*^
*f* −3 dB (high)	3110 Hz	3000 Hz^*^	3000^*^
Noise (input referred, max)	804 nV	800 nV	3 *μ*V
Power consumption of MEA surrounding electronics	550 mW	2 W	N.s.
Environment conditions	*T* 37°C, RH > 90%	*T* 37°C, RH < 60%	*T* 37°C, RH < 60%

^*^Specifications suggested to acquire spike signals (the bandwidth can be customized according to the user need). ^**^Standard value. N.s. = not specified. RH = relative humidity.
